# Ecology of Gene Drives: The Role of Density‐Dependent Feedbacks on the Efficacy and Dynamics of Two‐Locus Underdominance Gene Drive Systems

**DOI:** 10.1111/eva.70079

**Published:** 2025-03-06

**Authors:** Ziqian Xu, Michael B. Bonsall

**Affiliations:** ^1^ Mathematical Ecology Research Group, Department of Biology University of Oxford Oxford UK; ^2^ St Peters College Oxford UK

**Keywords:** density dependence, intraspecific competition, mathematical modelling, population dynamics, toxin‐antidote gene drives

## Abstract

Density dependence describes the regulation of population growth rate by population density. This process is widely observed in insect populations, including vectors such as mosquitoes and agricultural pests that are targets of genetic biocontrol using gene drive technologies. While there continues to be rapid advancement in gene drive molecular design, most studies prioritise gene drive efficacy over ecology, and the role of density‐dependent feedback on gene drives remains neglected. Furthermore, the details of density dependence experienced in these potential species of interest are usually poorly understood, creating additional constraints and challenges in evaluating the efficacy and efficiency of gene drive systems, especially those that promise local confinement after release. Here, we formulate and analyse a simple, non‐species‐specific mathematical model which integrates population dynamics by density dependence together with population genetics of a high‐threshold two‐locus underdominance system. Different models of density dependence and strengths of within‐species competition are investigated alongside other genetic and ecological parameters. Our results suggest that for an underdominance gene drive system, density dependence processes, by acting on births or deaths, influence the population dynamics by leading to significantly different population‐level suppression in the presence of a fitness cost. However, density dependence does not directly affect the fitness cost threshold for drive establishment. Moreover, we find that the magnitude and range of key ecological parameters (birth and death rates) could result in different outcomes depending on the type of density dependence employed. Our work highlights the importance of considering the ecological contexts in the design, development and deployment of gene drive molecular strategies.

## 1 Introduction

Synthetic gene drives are biologically engineered genetic constructs that can propagate throughout a population at a higher than Mendelian rate (Burt and Trivers 2006). These selfish genetic elements can spread through a population by biasing their own inheritance. Given these patterns of genetic spread, there is growing interest in deploying gene drives as a method for population control, such as targeting disease vectors, agricultural pests or invasive species, while concerns have also been raised over the safety of such technology (Esvelt et al. 2014; Oye et al. 2014).

From a regulatory perspective, the more desirable type of drive is the one that can achieve local confinement and is less likely to cause spillover (Marshall 2009). The past decade has witnessed the introduction of various ‘threshold’ drive systems which satisfy this property to remain confined: these systems, which self‐propagate by inducing selection against the wild‐type alleles in offspring, include *Medea* (Beeman, Friesen, and Denell 1992), engineered underdominance (Akbari et al. 2013; Davis, Bax, and Grewe 2001; Reeves et al. 2014), as well as more recent CRISPR‐based systems such as ClvR/TARE (Champer, Kim, et al. 2020; Oberhofer, Ivy, and Hay 2019). Such drive systems are sometimes broadly known as toxin‐antidote systems, as the drive construct typically consists of a toxin element that disrupts wild‐type functions and an antidote element that restores this disrupted function. Threshold dynamics are observed, as the toxin imposes a significant fitness disadvantage at low drive population frequencies, but it also gives the antidote a selective advantage at high drive population frequencies, where toxins are widespread (Marshall and Akbari 2018; Marshall and Hay 2012). While these proposed toxin‐antidote drives show promise as candidate strategies for genetic biocontrol, it remains unclear what roles different ecological conditions may play in affecting the efficacy of these threshold drive systems.

Specifically, density‐dependent intraspecific competition is widely observed in species as a negative feedback mechanism in population dynamics (Hanski 1990; Nicholson 1954; Sinclair 1989; Woiwod and Hanski 1992). Density dependence refers to the dependency of per capita growth rate of a population on its own density (Sinclair and Pech 1996). These sorts of non‐linear feedback mechanisms prevent populations from increasing exponentially when resources are limited and are important processes in determining patterns of competition and trophic interactions (Mueller and Joshi 2000; Turchin 2003). Investigating the interaction between the population genetics and ecological population dynamics of a gene drive is essential, as complex, non‐linear mechanisms may arise and in turn influence the persistence, dynamics and ecology of gene drive systems. Mathematical and ecological analyses have been conducted on the high‐threshold underdominance system to begin answering these questions (Edgington and Alphey 2017, 2018; Huang et al. 2011; Khamis et al. 2018; Magori and Gould 2006). However, as far as we are aware, no studies have yet considered the range of possible ecological outcomes of different density‐dependent processes affecting this high‐threshold gene drive system.

Previous work (Alphey and Bonsall 2014; Edgington and Alphey 2018) has explored only one specific density‐dependent function proposed by Maynard Smith and Slatkin (1973), as it was considered to be the best‐fitting model for describing the majority of population datasets analysed (Bellows 1981). However, this conclusion was based on the density‐dependent mortality data for insects available at the time, and exploring other forms of density dependence may provide a more comprehensive view for evaluating the interaction of density dependence with gene drive population genetics. This is especially important as little is known about the exact population dynamics of most gene drive target organisms such as pest species (Dhole, Lloyd, and Gould 2020).

Furthermore, density‐dependent processes result in very different behaviours depending on where and when they act in the life cycle. Thus far, density‐dependent models have only been assumed to operate on birth rates, but the effects of intraspecific competition on death rates can potentially lead to more significant population suppression (Dye 1984). Since our understanding of vector ecology remains largely absent amidst accelerating progress in molecular technologies, there is a growing imperative to investigate the range of intraspecific competition effects on the efficacy and efficiency of gene drive technologies.

Here, we explore the effects of different types of density‐dependent processes acting on either births or deaths and their effects on the efficacy and ecological dynamics of an underdominance gene drive system. This study is an expansion of our previous work (Alphey and Bonsall 2014; Khamis et al. 2018) and subsequent studies (Edgington and Alphey 2017, 2018) by adapting Kostitzin's framework (Kostitzin 1939) that employed Lotka‐Volterra style competition equations to categorise different genotypes. Specifically, we investigate the role of ecological intraspecific processes on high‐threshold synthetic two‐locus underdominance constructs. We begin by introducing the mathematical models and details of the analysis undertaken. Next, we analyse the influence of different nonlinear intraspecific competition models on the performance of two‐locus underdominance gene drives. Finally, we consider the importance of our findings in light of recent development of gene drives and their ecological implications.

## 2 Methods

**FIGURE 1:**
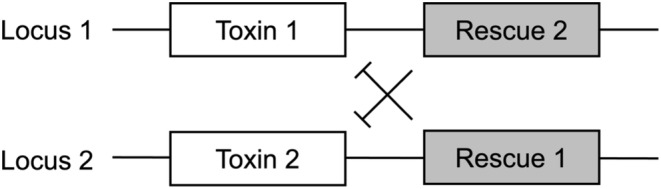
A schematic diagram of the two‐locus underdominance gene drive system. The drive at locus 1 contains a toxin that targets locus 2 for cleavage. Simultaneously, the drive at locus 2 contains a rescue gene which suppresses this toxin. The same applies to the toxin at locus 2, which is suppressed by the rescue gene at locus 1. Thus, both or neither drives must be present for survival. Both constructs may contain additional cargo for introducing desirable traits (not shown in this figure).

Here, we focus on a two‐locus underdominance gene drive system. Underdominance has a well‐established record of being a prospective candidate for biocontrol (Curtis 1968). Whilst sharing some design principles with its precursors, such as cytoplasmic incompatibility (Curtis and Adak 1974) and *Medea* (Beeman, Friesen, and Denell 1992), this engineered gene drive system was first proposed in theory by Davis and co‐workers (Davis, Bax, and Grewe 2001). It is designed in such a way that the first drive construct targets the first locus (toxin) while providing rescue to a second locus (antidote) and vice versa (Figure 1). In order to survive, develop and reproduce, individuals must carry at least one drive copy (so‐called haplosufficiency) at both loci for survival, unless they are wild types. The two‐locus underdominance system belongs to the category of high‐threshold drives, as there is an initial release threshold even in the absence of a fitness cost.

In our model, this system comprises two genetically unlinked loci, giving rise to nine different genotypes. These genotypes are denoted by a four‐letter notation, as presented in previous studies (Champer, Zhao, et al. 2020; Edgington and Alphey 2018; Khamis et al. 2018), where A & B represent drive alleles, and a & b represent wild‐type alleles at these two loci (e.g., wild types are aabb, and AaBb represents heterozygosity at both loci).

For population birth rate, the densities of individuals *N*_*i*_ are replaced with progeny *ν*_*i*_, which are functions of the mating crosses of nine genotypes involved (detailed in Appendix S1). The mathematical model presented here is deterministic and continuous. A panmictic (random‐mating) population is assumed in this model with no genetic drift or genetic mutation. State variables and parameters are presented in Table 1.

**TABLE 1:** Summary of symbols, state variables and parameters.

Symbol	Description	Value	Comments
*N*_*i*_(*t*)	Number of individuals of genotype *i*	—	State variables of the model
*N*(*t*)	Number of total individuals	—	—
*ν*_*i*_	Number of offspring of genotype *i* as a result of dihybrid crosses	See Appendix II: S1	The genotype frequencies from dihybrid crosses are presented in Appendix I: S1
*r*	Population birth rate	1.1	Unit per day. Approximation of previously used value in Alphey and Bonsall (2014) for *Anopheles* mosquitoes
*μ*	Population death rate	0.1	Unit per day. Approximation of previously used value in Alphey and Bonsall (2014) for *Anopheles* mosquitoes
*a*	Density parameter for scaling the population size	See Appendix IV: S1	Calculated to produce a natural population equilibrium of 10,000
*b*	Strength of density‐dependent competition	0.5, 2	*b* < 1 indicates undercompensation, *b* = 1 indicates perfect compensation, *b* > 1 indicates overcompensation
*s*	Relative fitness cost due to construct	0 ≤ *s* ≤ 1	0 (no penalty) to 1 (lethal)
*h*	Dominance of fitness cost in heterozygotes	0 ≤ *h* ≤ 1	0 (recessive) to 1 (dominant)
*ψ*_*i*_	Relative fitness cost of genotype *i*	See Table 3	—

*Note:* The density (numbers per unit area) of individuals of the nine genotypes, *i*, is denoted by *N*_*i*_ (Table 3). As we consider population densities, the values of *N*_*i*_ here can be any real number. The population birth rate *r* and death rate *μ* are assumed to be constant for all genotypes.

A summary of the density‐dependent models employed is provided in Table 2. Two versions of the model were formulated based on which parameter the density‐dependent intraspecific competition functions act upon:

1. Density‐dependent births: where intraspecific competition $f\left(N\right)$ acts on the birth rate while the death rate remains constant. Density‐dependent birth functions have an initial value of one, which decreases to zero as the population grows. The general form is
$$\frac{dN_{i}}{\mathrm{dt}}=r\nu_{i}\left(t\right)\psi_{i}f\left(N\right)-\mu N_{i}\left(t\right)$$
2. Density‐dependent deaths: where intraspecific competition $f\left(N\right)$ acts as an additional effect on the death rate while the birth rate remains constant. Density‐dependent functions have an initial value of zero, which increases to (but does not stop at) one as the population grows to equilibrium. This function is treated as an addition rather than a multiple of the baseline death rate *μ*. This ensures that a death rate would still be present when the population is indefinitely small. The general form is
$$\frac{dN_{i}}{\mathrm{dt}}=r\nu_{i}\left(t\right)\psi_{i}-\left(\mu +f\left(N\right)\right)N_{i}\left(t\right)$$

**TABLE 2:** Summary of density‐dependent models.

Authors	Density‐dependent births	Density‐dependent deaths
Maynard Smith and Slatkin (1973)	(1 + (*aN*)^*b*^)^−1^	ln(1 + (*aN*)^*b*^)
Hassell (1975)	(1 + *aN*)^−*b*^	*b*ln(1 + *aN*)
Bellows (1981)	exp(–*aN* ^*b*^)	*aN* ^*b*^

*Note:* Types of density‐dependent functions as outlined by Bellows's review, in their corresponding forms of density dependence, where *b* represents the strength of density dependence. aN in this table denotes the total population of all genotypes, i.e. $\sum_{j=1}^{9}a\nu_{j}\left(t\right)$ in the models.

A more comprehensive presentation of the full mathematical model for each density‐dependent model is provided in the Appendix S1.

Relative fitness costs of the drive always act on population births and are accounted for as follows: the fitness of wild types is assumed to be one at both loci. Drive homozygotes carry a fitness penalty, *s*, as a result of the novel drive construct ranging from 0 (as fit as wild types) to 1 (non‐viable). Heterozygotes also have an additional factor, *h*, which determines the dominance of fitness costs ranging from 0 (recessive) to 1 (dominant). Fitness parameters are assumed to be independent at each locus, resulting in two pairs: *s*_*a*_, *s*_*b*_ and *h*_*a*_, *h*_*b*_. The six types of gametes hence have the fitness as follows: *ψ*_*aa*_ = 1, *ψ*_*Aa*_ = (1 − *h*_*a*_ *s*_*a*_), *ψ*_*AA*_ = (1 − *s*_*a*_); *ψ*_*bb*_ = 1, *ψ*_*Bb*_ = (1 − *h*_*b*_ *s*_*b*_), *ψ*_*BB*_ = (1 − *s*_*b*_). In addition, complete penetrance (haplosufficiency) is assumed from toxins and antidotes of the two‐locus underdominance system (one copy of toxin is sufficient to suppress all offspring, and one copy of the rescue gene is sufficient to inhibit any toxin). Hence, any non‐wild‐type genotypes without at least one drive element at both loci are deemed non‐viable (in this study, genotypes AAbb, Aabb, aaBB and aaBb) and are assigned a relative fitness of zero. For the remaining genotypes, this study assumes multiplicative effects of fitness costs from the two loci in determining their relative fitness, as presented in Table 3. This assumption ensures all genotypes have a fitness value between 0 and 1.

**TABLE 3:** Notations, lethality and relative fitness of nine genotypes.

*N*_*i*_	Genotype	Lethal effects	Relative fitness *ψ*_*i*_
*N*_1_	*AABB*	No	(1 − *s*_*a*_)(1 − *s*_*b*_)
*N*_2_	*AABb*	No	(1 − *s*_*a*_)(1 − *h*_*b*_ *s*_*b*_)
*N*_3_	*AAbb*	Yes	0
*N*_4_	*AaBB*	No	(1 − *h*_*a*_ *s*_*a*_)(1 − *s*_*b*_)
*N*_5_	*AaBb*	No	(1 − *h*_*a*_ *s*_*a*_)(1 − *h*_*b*_ *s*_*b*_)
*N*_6_	*Aabb*	Yes	0
*N*_7_	*aaBB*	Yes	0
*N*_8_	*aaBb*	Yes	0
*N*_9_	*aabb*	No	1

*Note:* Genotypes are numbered from 1 (drive homozygote) to 9 (wild types). Relative fitness is calculated by multiplying independent fitness at each locus while considering the viability of each genotype.

In contrast to previous work, the model is generic and hence does not incorporate life stage structures of any particular species. Nevertheless, some parameters used in the study, such as the birth rate *r* and the death rate *μ*, are approximately based on those of *An. gambiae* mosquitoes. This approach ensures our work provides a reasonable comparison with previous literature while keeping the model as simple, generic and straightforward as possible for investigating the different effects of density dependence on underdominance gene drives.

Since the model operates in continuous time, all parameters are expected to take effect simultaneously. Thus, fitness costs and density‐dependent processes are assumed to be independent of each other. This again contrasts with earlier work, where fitness cost may be able to act on density‐dependent functions, depending on which stage fitness costs take effect (referred to as early‐ or late‐acting fitness costs in the literature).

Previous population‐level models investigating gene drive dynamics have focused on a single density‐dependent intraspecific process. Here, the aim is to explore a range of density‐dependent intraspecific functions (Bellows 1981). These models employed include three different functions and are outlined in Table 2: the two‐parameter density‐dependent functions proposed by Maynard Smith and Slatkin (1973) (abbreviated as MSS hereinafter), Hassell (1975) and Bellows (1981). When the strength of density dependence is perfectly compensating (*b* = 1), the MSS and Hassell models have the same form. This form was previously proposed by Skellam (1951). Each model of density dependence can then give rise to two forms depending on whether they act on the birth or death rate, as previously described.

All analyses and simulations were carried out in R. We used fourth‐order Runge–Kutta methods to obtain numerical solutions of the differential equations for the population dynamics. During each simulation, the population was first established with a wild‐type population at a stable equilibrium of 10,000 individuals for 10 generations to ensure accurate numerical integrations. Drive homozygotes were then released in a 1:1 ratio with respect to the wild‐type population density and the model was simulated for a further 200 generations. This 1:1 release ratio is significantly larger than the introduction threshold frequency previously determined for two‐locus underdominance drives (Champer, Zhao, et al. 2020), ensuring that the drive could successfully spread in the absence of a fitness cost. Models were run, with different ecological parameters as independent variables, to determine (after 200 generations): (i) final total population density, (ii) final drive‐carrying population density and (iii) time taken for wild types to *return* to the equilibrium when the gene drive failed to reach fixation. As populations are expressed in terms of densities, here *return* is arbitrarily defined as achieved when the population exceeds 99% of the population at equilibrium (i.e., 9,900 individuals). Furthermore, three strengths of density dependence are considered for each density‐dependent model in all simulations: *b* = 0.5 (undercompensating), *b* = 1.0 (perfectly compensating) and *b* = 2.0 (overcompensating). The dominance of fitness costs in heterozygotes, *h*_*a*_ and *h*_*b*_, is assumed to be equal for all simulations (hereafter referred to jointly as *h*).

The scripts are available at: https://osf.io/strhu/.

We begin our analysis by considering a simplified case where relative fitness costs of each locus are assumed to be equal (*s*_*a*_ = *s*_*b*_). First, the effects of different density‐dependent functions and varying fitness costs on the efficacy of gene drive (in terms of total population density, drive‐carrying population density and wild‐type recovery time) were explored. Simulations were carried out over the whole range of fitness costs (*s*), from 0 to 1, while dominance of fitness cost (*h*) is fixed at 0.5. Second, the effects of different density‐dependent functions and varying dominance of fitness cost on heterozygotes on gene drive efficacy were explored, for the whole range of *h* from 0 to 1. The fitness cost (*s*) was fixed at 0.23, a point close to the fitness cost threshold for gene drive establishment and hence an ideal point to investigate the effects of *h*, as such costs become significant only when the fitness costs (*s*) are large enough.

Next, we considered the case when fitness costs for loci A and B are not equal. For simplicity, it was assumed that *s*_*b*_ is always equal to or greater than *s*_*a*_, and the ratio between the two fitness costs (*s*_*b*_/*s*_*a*_), ranges from 1 to 2, with 11 sets of simulations performed across this range with an increment of 0.1. We explored the effects of density‐dependent models and fitness costs on drive efficacy, using a heatmap to illustrate the total population percentage relative to the equilibrium size (10,000), with *s*_*a*_ ranging from 0 to 0.5 on the *x*‐axis and the ratio *s*_*b*_/*s*_*a*_ on the *y*‐axis. The range of *s*_*a*_ only goes up to 0.5 to ensure that *s*_*b*_ does not exceed 1. Heatmaps for all models were generated with *h* fixed at 0.5, and later for the undercompensating MSS model when under three scenarios: *h* = 1 (dominant), *h* = 0.5 (default), and *h* = 0 (recessive).

Finally, to highlight the importance of selecting appropriate ecological values and the potential impact on the outcome of drive establishment from ecological anomalies, we conducted sensitivity analyses by examining the effects of fitness costs and density‐dependent interactions on drive efficacy (with equal fitness costs at two loci). We further considered the following ecological conditions: (i) control scenario where both values are at default, when birth rate *r* = 1.1 and death rate *μ* = 0.1, (ii) high birth rate scenario, when birth rate *r* is increased to 2.1 while death rate remains at 0.1 and (iii) high death rate scenario, where death rate is increased to 0.2 while birth rate remains at 1.1. Based on the default birth rate and death rates we selected, the per capita growth rate always equals 1 initially when the density is infinitely small and always equals 0 when the population reaches its equilibrium. However, the initial fixed point at 1 is no longer guaranteed under other scenarios as it is subject to changes in birth or death rates.

## 3 Results

### 3.1 How Do the Density‐Dependent Functions Compare in Terms of Effects on Per Capita Growth Rate?

**FIGURE 2:**
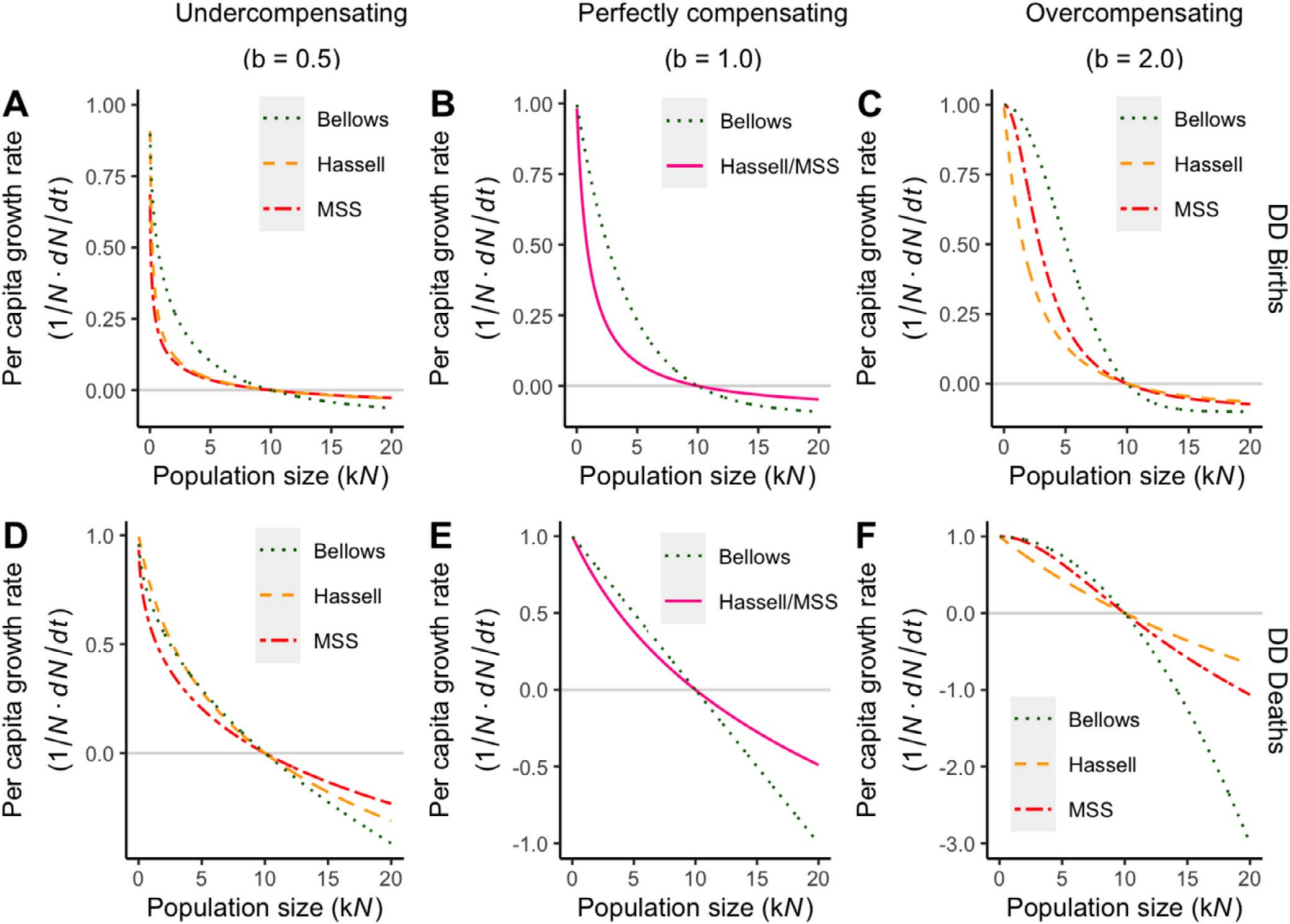
Comparison of density‐dependent models for births and deaths in terms of per capita growth rate. Density‐dependent feedback models from Table 2, as previously described in Bellows (1981), are presented here. The strength of density‐dependent regulation for each model is plotted against the growing population density while the equilibrium is set at 10,000 individuals. The density‐dependent function on birth rates is a scaling factor to the birth rate *r*, which starts at 1 and decreases to 0. The density‐dependent function on death rates is an addition to the death rate *μ*, which starts at 0, increases with population density and reaches 1 at equilibrium. Per capita growth rates are calculated by incorporating these functions to obtain the difference in regulated birth rate *r*′ and death rate *μ* in density‐dependent births and between the birth rate *r* and regulated death rate *μ*′ in density‐dependent deaths. The per capita growth rate is at 1 initially and decreases to 0 as the population grows to equilibrium at 10,000. Each model is compared against one another when the strength of density dependence is: (A, D) *b* = 0.5, undercompensating, (B, E) *b* = 1, perfectly compensating, (C, F) *b* = 2, overcompensating and when the density dependence acts on (A–C) births and (D–F) deaths. When the models are perfectly compensating (*b* = 1), the MSS and Hassell models display the same form. A grey *y*‐intercept at zero is included in each figure.

We begin by investigating the influences of three density‐dependent functions on per capita growth rates. The per capita growth measures changes in population size, scaled by the current population density, between successive time points. Here we aim to compare how the per capita growth rate of each model changes in relation to population density under different strengths of competition (undercompensating, perfectly compensating and overcompensating). Illustrations of the results are shown in Figure 2.

First, we consider perfectly compensating population dynamics (*b* = 1.0) for the Bellows and Hassell/MSS models. In density‐dependent births, both models result in concave‐up shaped density dependence with per capita growth rates below 0.25 when the population reaches half of the equilibrium size (Figure 2B). The perfectly compensating Hassell/MSS model leads to a faster reduction in per capita growth rate than the Bellows model below equilibrium. Under density‐dependent deaths (Figure 2E), the Bellows function is negatively linear. The perfectly compensating Hassell/MSS function is logarithmic and again leads to a more significant decrease in net growth rate than the Bellows function until population size exceeds the equilibrium. However, the decrease in growth for both types of models under this condition is more gradual compared to density‐dependent births.

When the population dynamics are no longer assumed to be perfectly compensating, MSS and Hassell models begin to show differences, with the regulatory effects also dependent on the strength of competition. In general, an undercompensating strength of density dependence would lead to sharper reductions in per capita growth rates when the population is small, as the weaker undercompensating density dependence is less able to respond to changes in density.

In undercompensating dynamics (*b* = 0.5), Hassell and MSS functions display similar effects on growth below the population equilibrium in density‐dependent births, with net growth rate values of less than 0.1 at half of the equilibrium size. The density‐dependent feedback produced by the MSS function results in marginally lower net growth rates, while Bellows function has the most gradual effect on decreasing net growth rate under this condition (Figure 2A). In contrast, the MSS/Hassell functions differ more significantly when density dependence is acting on deaths: whilst the MSS function is still observed as the model producing the lowest net growth rate when the population is small, changes in growth in all models are more moderate within the range relative to density‐dependent births, but they remain as concave‐up curves (with per capita growth rates at around 0.3 at half of equilibrium). The Hassell model, meanwhile, has effects on net growth rate similar to that of Bellows when population density is smaller than the equilibrium (Figure 2D).

When the population dynamics are overcompensating (*b* = 2.0), the three models are sigmoidal in density‐dependent births, with Hassell function resulting in the lowest and Bellows function leading to the highest net growth rates below the population equilibrium (growth rates at 0.12 and 0.5 at half of the equilibrium, respectively; Figure 2C). Under density‐dependent deaths, both MSS and Bellows models exhibit concave‐down curves, while the Hassell function is almost negatively linear (Figure 2F).

### 3.2 How Do the Fitness Cost and Density Dependence Interact to Affect Gene Drive Efficacy?

**FIGURE 3:**
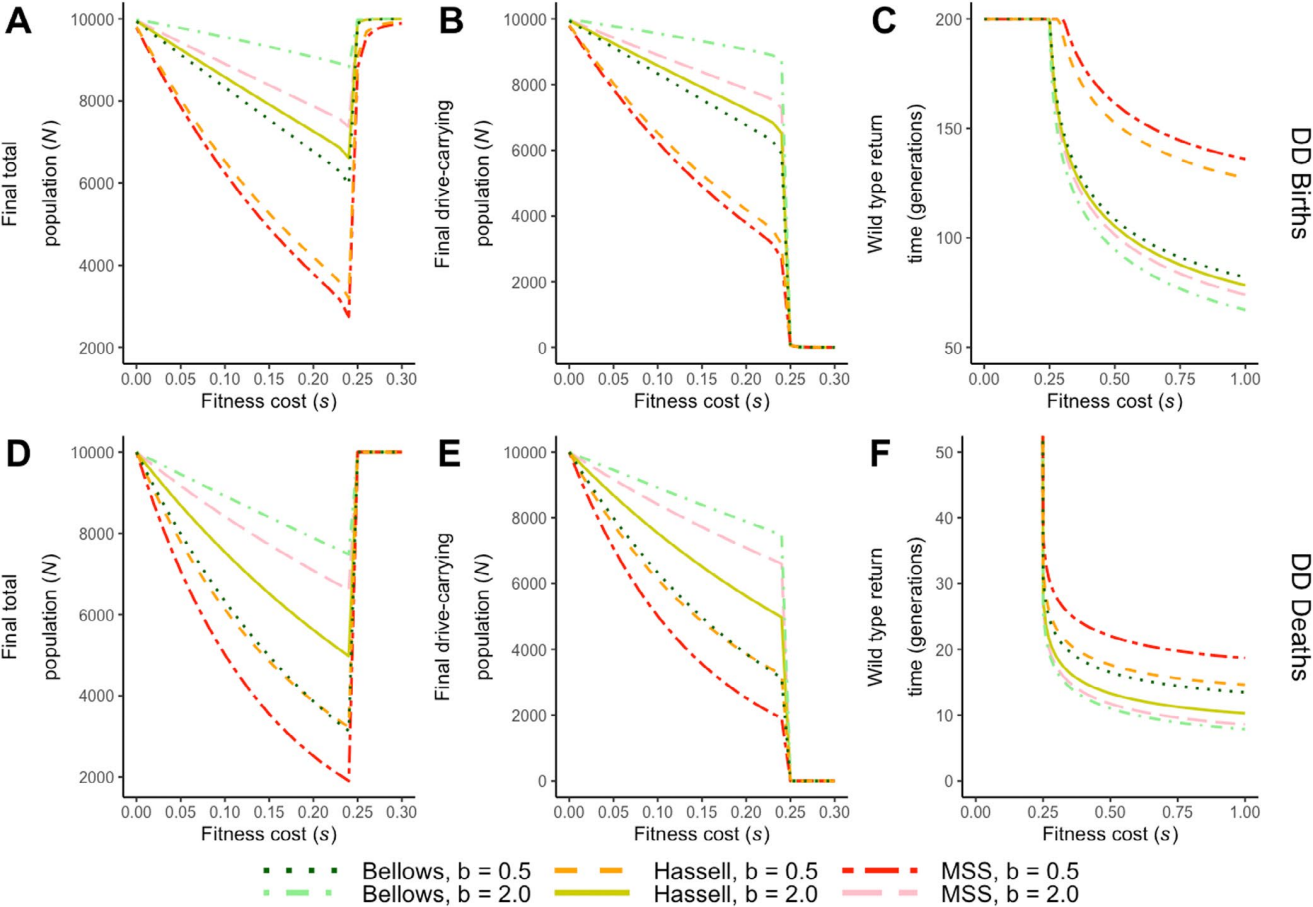
The effects of varying fitness cost (*s*) and density dependence on gene drive efficacy. Results are illustrated in terms of (A, D) the final total population size 200 generations after drive release, (B, E) the final drive‐carrying population size 200 generations after drive release and (C, F) the number of generations required for wild types to return (defined as reaching above 99% of equilibrium) and when the density dependence acts on (A–C) births and (D–F) deaths. Density‐dependent functions were simulated under two strengths: Undercompensating (*b* = 0.5) and overcompensating (*b* = 2.0). Simulations were run across the full range of *s* from 0 to 1, with an increment of 0.01, but results are displayed only up to 0.30 for (A, B, D and E), as the drive is always lost from the population beyond this point. The range for wild‐type return time is different for (C and F).

As expected, a fitness threshold is observed for two‐locus underdominance gene drives. This is the case when the population is subject to both the density‐dependent birth and death processes (Figure 3). Below this threshold (a value observed at ~0.24–0.25), the population declines from its equilibrium due to the fitness cost imposed on drive constructs (Figure 3A,D). This occurs as the drives become successfully established and are fixed in the population at the end of each simulation, a process suggested by the corresponding population densities of drive‐carrying genotypes and that of all individuals (Figure 3B,E). Consequently, increasing the fitness penalty for the drive leads to greater suppression for the entire population, as it predominantly consists of drive‐carrying individuals.

On the other hand, the level of population suppression observed is determined crucially by the details of density‐dependent regulations, such as the type of models employed, and the strength of intraspecific competition. Differences are also observed between the timing of density‐dependent processes, where the density decline is approximately linear under density‐dependent births, but concave‐up with more significant density suppression in models of density‐dependent deaths.

When fitness cost exceeds the threshold, drive constructs fail to achieve establishment and are eventually lost. This occurs as the fitness cost for the drive construct increases the introduction threshold frequency required for drive establishment. In our model, this drive would fail if the high fitness cost resulted in an invasion frequency exceeding our default setting of 1:1 drive release to wild‐type ratio.

Meanwhile, the wild‐type population recovers back to the equilibrium, and the time taken for wild types to return is inversely related to the fitness cost and additionally affected by the type of density‐dependent regulations. Wild‐type recovery time is significantly more rapid under density‐dependent deaths, typically within 20 generations without a fitness cost, compared to up to 150 generations under density‐dependent births (Figure 3C,F).

#### 3.2.1 The Role of Density‐Dependent Births on Underdominance Gene Drive Dynamics

To investigate the role of density dependence on births, we begin by looking at different density‐dependent birth processes when they are subject to weak, undercompensating dynamics (*b* = 0.5). Greater population suppression due to increasing fitness cost was observed for the two closely related two‐parameter models (MSS and Hassell) compared to the Bellows model. Under these density‐dependent models, the population is reduced to nearly 25% of the original equilibrium level, up to the fitness threshold, with the longest wild‐type recovery times occurring beyond this threshold. Amongst these two related functions, the MSS model showed higher levels of population reduction and longer recovery times for wild types (Figure 3A–C). In contrast, with overcompensating dynamics (*b* = 2.0), a greater reduction in population was observed in the Hassell density dependence compared to MSS, while both models showed greater population suppression, leaving down to 70% of individuals at the fitness threshold compared to around 90% under the Bellows density dependence.

These effects can be explained by comparing the outcomes to the per capita growth rate results (Figure 2A,C). Density dependence in undercompensating MSS/Hassell models results in a smaller net growth rate compared to the Bellows model below the equilibrium. As density quickly decreases below equilibrium due to transgenic release and density‐dependent processes, density dependence in MSS/Hassell models is less likely to counteract the suppressive effects on the population caused by stronger fitness penalties. Similarly, this explains why the greatest population suppression occurs in the MSS model under undercompensating dynamics and in the Hassell model under overcompensating dynamics, as these models produce the lowest per capita growth rates below equilibrium under their respective conditions.

Overall, undercompensating feedback is weaker and reduces the population's ability to respond. This leads to a greater reduction in the population size of drive‐carrying genotypes and extends the recovery time for wild types compared to overcompensating feedback. On the other hand, overcompensating dynamics provides a stronger feedback, which offsets the suppressive effects from gene drive genetics (Figure 3A,C). Furthermore, the MSS function is more sensitive to variations in the strength of density dependence (*b*), as populations regulated by MSS experience greater changes in size compared to those regulated by Hassell.

#### 3.2.2 The Role of Density‐Dependent Deaths on Underdominance Gene Drive Dynamics

Next, the efficacy of the underdominance gene drive system under density dependence acting on death rate is investigated. With undercompensating dynamics (*b* = 0.5), the most suppressive effects on population were found in the density‐dependent process described by the MSS function, with only 20% of the population left around the fitness threshold value and also having the longest time for wild types to recover if drives failed to establish. The Hassell and Bellows models now result in similar levels of population decline and a population reduction of 70% (3000 individuals left). In particular, the population dynamics of the Bellows function observed here significantly differ from its results on density‐dependent births, where the total population is at 60% of the population equilibrium (6000 individuals) below the fitness threshold and more comparable to other overcompensating models.

For overcompensating dynamics (*b* = 2.0), the most significant decrease in population was again observed in the Hassell model, with around half of the population suppressed at maximum fitness cost below the threshold, and the longest recovery time for wild types among the three models above the fitness threshold. This is followed by the MSS function (less than 40% suppressed) and the Bellows function (less than 30% suppressed). These findings square with the per capita growth rates under density‐dependent deaths in Figure 2, where MSS has the lowest undercompensating net rate of growth and Hassell has the lowest overcompensating rate at low population levels.

### 3.3 How Do the Dominance of Fitness Cost and Density Dependence Interact to Affect Gene Drive Efficacy?

**FIGURE 4:**
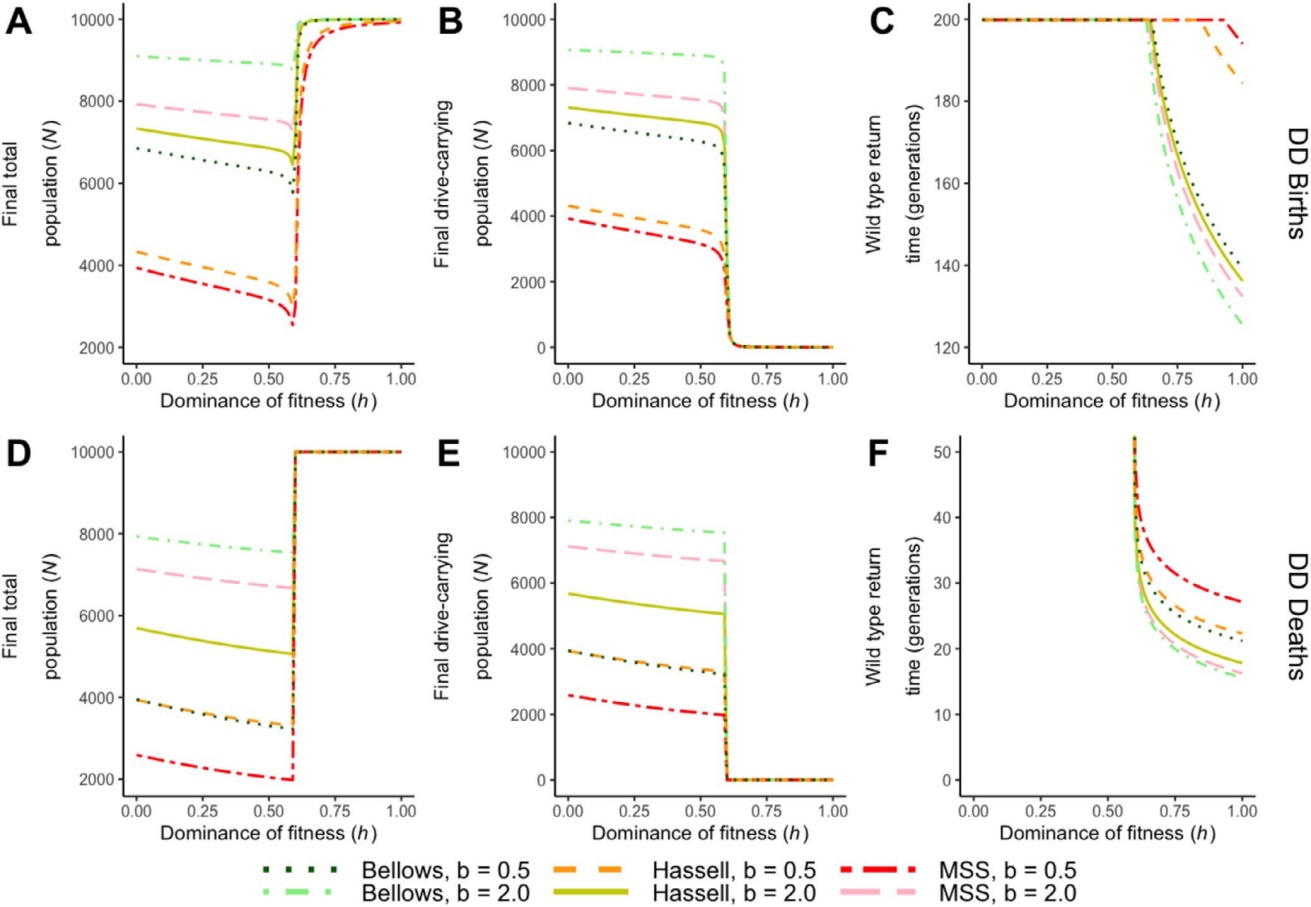
The effects of varying dominance of fitness cost in heterozygotes (*h*) and density dependence on gene drive efficacy. Results are illustrated in terms of (A, D) the final total population size 200 generations after drive release, (B, E) the final drive‐carrying population size 200 generations after drive release and (C, F) the number of generations required for wild types to return (defined as reaching above 99% of equilibrium) and when the density dependence acts on (A–C) births and (D–F) deaths. Density‐dependent functions were simulated under two strengths: Undercompensating (*b* = 0.5) and overcompensating (*b* = 2.0). Simulations were run across the full range of *h* from 0 to 1, with an increment of 0.01. The fitness cost (*s*) is set at 0.23 for this analysis, which is a point close to the fitness cost threshold. The range for wild‐type return time is different for (C and F).

Results investigating the effects of the dominance of fitness costs (*h*) on drive efficacy reveal the presence of a threshold value for *h* (Figure 4), similar to the threshold observed for the fitness cost (*s*). Below this threshold (a value observed between 0.59 and 0.60), the population declines in size due to drive fixation, with increasing fitness penalties imposed on drive heterozygotes. When *h* surpasses this threshold, the gene drive is lost, and the wild‐type population recovers, with the time taken for this process inversely proportional to the value of *h*.

Regardless of whether density dependence affects births or deaths, it remains the undercompensating MSS type of density dependence that exhibits the greatest reduction in population size below the threshold and the longest wild‐type recovery time above the threshold. In contrast, the overcompensating Bellows model shows the least reduction and the quickest return time (Figure 3; when *s* = 0.23). The effects of the dominance of fitness cost on the level of population reduction produced below the threshold are almost linear, with a similar gradient across all type of density dependence, under either under‐ or overcompensating dynamics (around 1000 individuals are lost as *h* increases from 0 to the threshold).

Notably, this threshold for *h* is closely related to, and influenced by, the value of *s*. For example, if s is fixed at 0.05, no threshold for *h* would be present, as the drive is expected to reach fixation even when heterozygotes carry the maximum penalty on fitness. However, when the fitness cost is at or near the threshold value, the dominance of fitness (*h*) can play an important role in determining the fate of a drive release (Figure 4).

### 3.4 How Do the Fitness Cost and Density‐Dependent Mortality Interact to Affect Gene Drive Efficacy When Fitness Costs at the Two Loci Are Asymmetrical?

In this section, equal fitness costs at loci A and B are no longer assumed for the drive construct. Following the preceding analysis, the fitness ratio between the two loci (*s*_*b*_/*s*_*a*_, as detailed in Methods) is incorporated as a new measure for fitness cost on total population size. Only results for density‐dependent death models are discussed in this section, as density‐dependent birth models yield similar results (see Figure S1).

**FIGURE 5:**
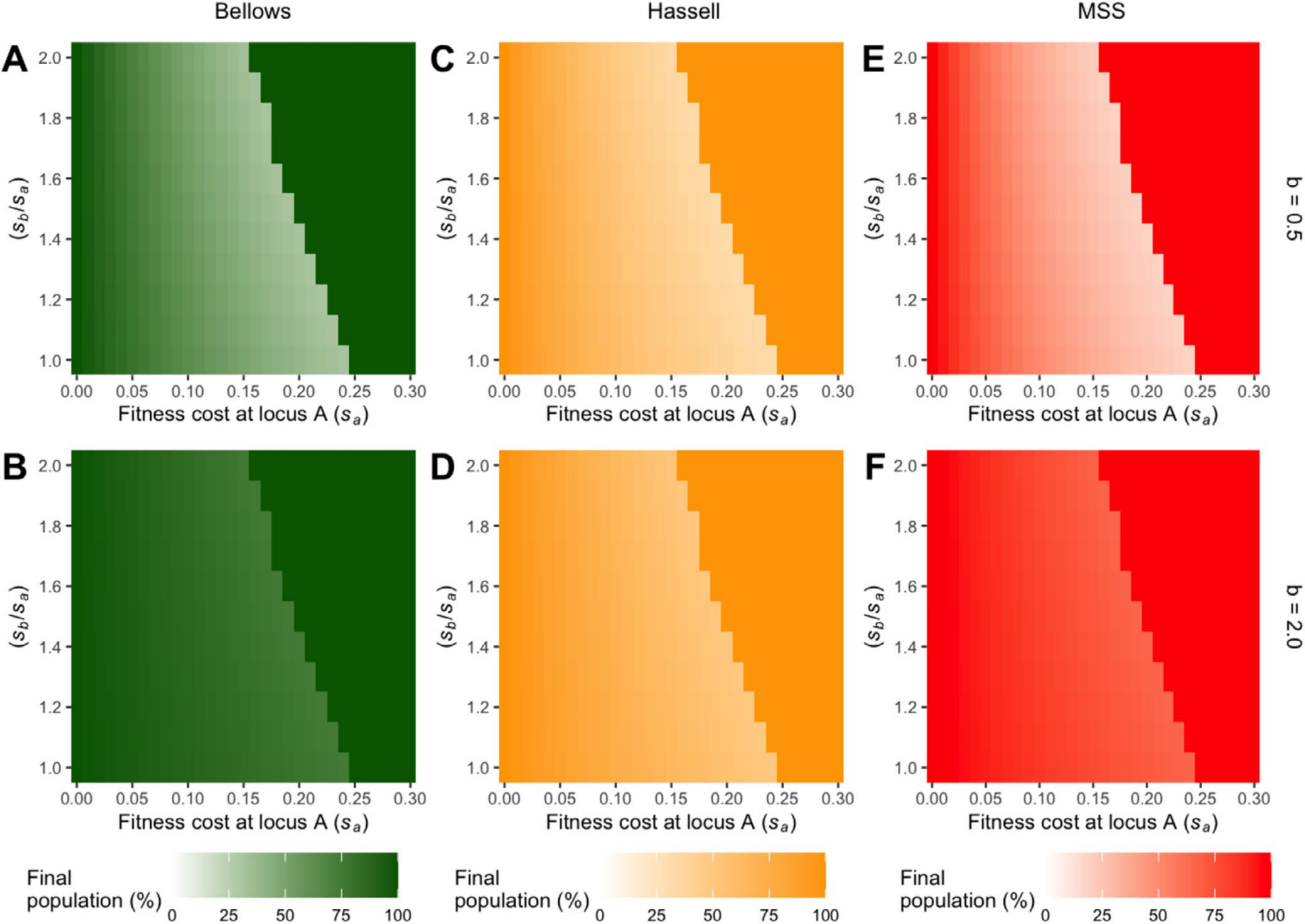
Heatmaps for drive efficacy analysis of varying fitness costs (*s*) of density‐dependent death models without assuming equal fitness costs at the two loci. The fitness cost ratio between the two loci (*s*_*b*_/*s*_*a*_) is plotted against the fitness cost at locus A (*s*_*a*_), while the final total population percentage is indicated by the colour gradient: Minima are coloured in white, while maxima are coloured in green, orange and red for Bellows (A, B), Hassell (C, D) and MSS (E, F) models, respectively. The top panel (A, C, E) is for undercompensating dynamics at *b* = 0.5, while the bottom panel (B, D, F) is for overcompensating dynamics at *b* = 2.0. Simulations were run across the full range of *s*_*a*_ from 0 to 0.5, with an increment of 0.01, but results are displayed only up to 0.30 in the figures.

In summary, heatmaps generated display similar features across the different types of density dependence employed (Figure 5). Results for undercompensating dynamics in general, as expected from the previous analysis, result in more significant population suppression by the increase in fitness costs. However, all results share similar inherent characteristics in terms of fitness threshold boundaries compared to the results for overcompensating density dependence.

Importantly, as characterised by these common fitness threshold boundaries, an increase in the fitness ratio *s*_*b*_/*s*_*a*_ between the two loci reduces the threshold fitness costs required for the drive to achieve fixation. For instance, in any of the density‐dependent death models, a fitness cost of 0.20 would allow the drive to establish if *s*_*b*_ = *s*_*a*_, but the drive would be lost if *s*_*b*_ = 2*s*_*a*_. This decline in threshold fitness cost due to increasing fitness ratios at the two loci is predominantly linear (Figure 5).

#### 3.4.1 Additional Effects From the Dominance of Fitness Cost on Fitness Threshold for Drive Efficacy on Heatmaps

As neither the type nor the strength of density dependence affects the fitness cost threshold boundaries, we further explore the interplay between the dominance of fitness cost in heterozygotes (*h*) and fitness cost (*s*) in determining the fitness threshold. Results for undercompensating MSS models with density‐dependent birth or death processes are investigated here with three different values of *h* (0, 0.5, and 1). Similar results for Bellows and Hassell types of density dependence are given in Figure S2 (undercompensating) and Figure S3 (overcompensating).

**FIGURE 6:**
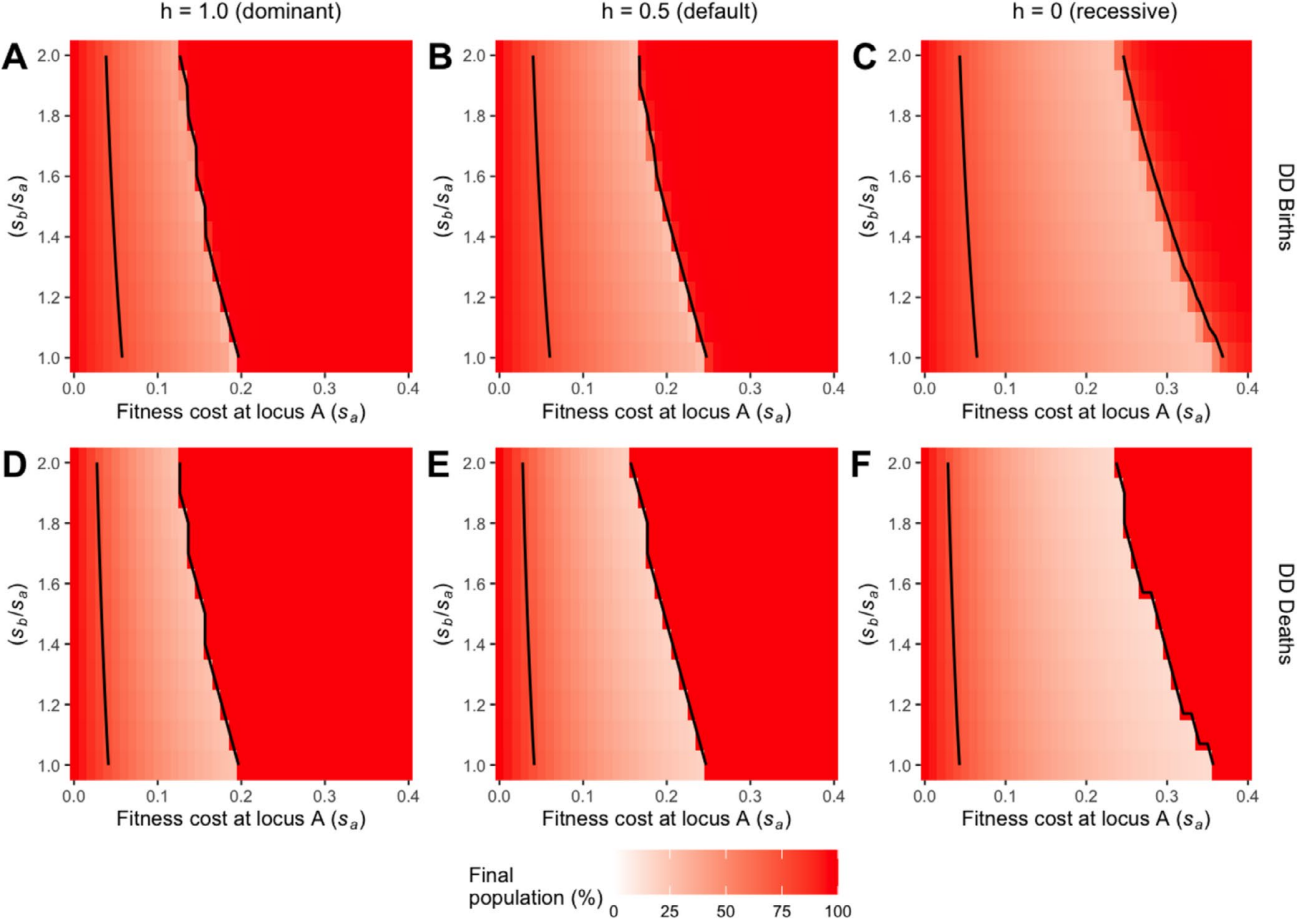
Heatmaps for drive efficacy analysis of varying fitness costs (*s*) of undercompensating density‐dependent MSS models in three conditions of dominance of fitness cost (*h*) without assuming equal fitness costs at two loci. Here, heatmaps in Figure 5 are performed under three scenarios of dominance of fitness costs in heterozygotes (*h*): (A, D) when *h* is fully dominant at 1.0, (B, E) when *h* is default at 0.5, (C, F) when *h* is fully recessive at 0, and when the density dependence acts on (A–C) births and (D–F) deaths. The percentage of the final total population is indicated by the colour gradient where minima are coloured in white and maxima are coloured in red. Contour lines are drawn in black to delineate boundary areas using an arbitrary threshold percentage of 0.75. Simulations were run across the full range of *s*_*a*_ from 0 to 0.5, with an increment of 0.01, but results are displayed only up to 0.40 in the figures.

As the dominance of fitness cost in heterozygotes (*h*) increases from 0 to 1, the fitness cost threshold is reduced, narrowing the fitness window for successful drive establishment (Figure 6). This result aligns with previous analyses (see Figure 4), where a high value of *h* could result in failed drive establishment that would otherwise succeed if *h* was lower. Additionally, this analysis further demonstrates the range of fitness cost within which *h* could generate additional threshold conditions for drive persistence or failure.

### 3.5 How Do Ecological Parameters and Density Dependence Interact to Affect Gene Drive Efficacy?

In this final section, we explore the sensitivity of gene‐driven dynamics to alterations in a wider range of birth and death rates and investigate how results would differ from the default parameters used in Figure 3. Specifically, we assess the wild‐type recovery time to the original equilibrium as a measure of drive efficacy.

#### 3.5.1 Density‐Dependent Births: The Sensitivity of Ecological Parameters on Drive Efficacy

**FIGURE 7:**
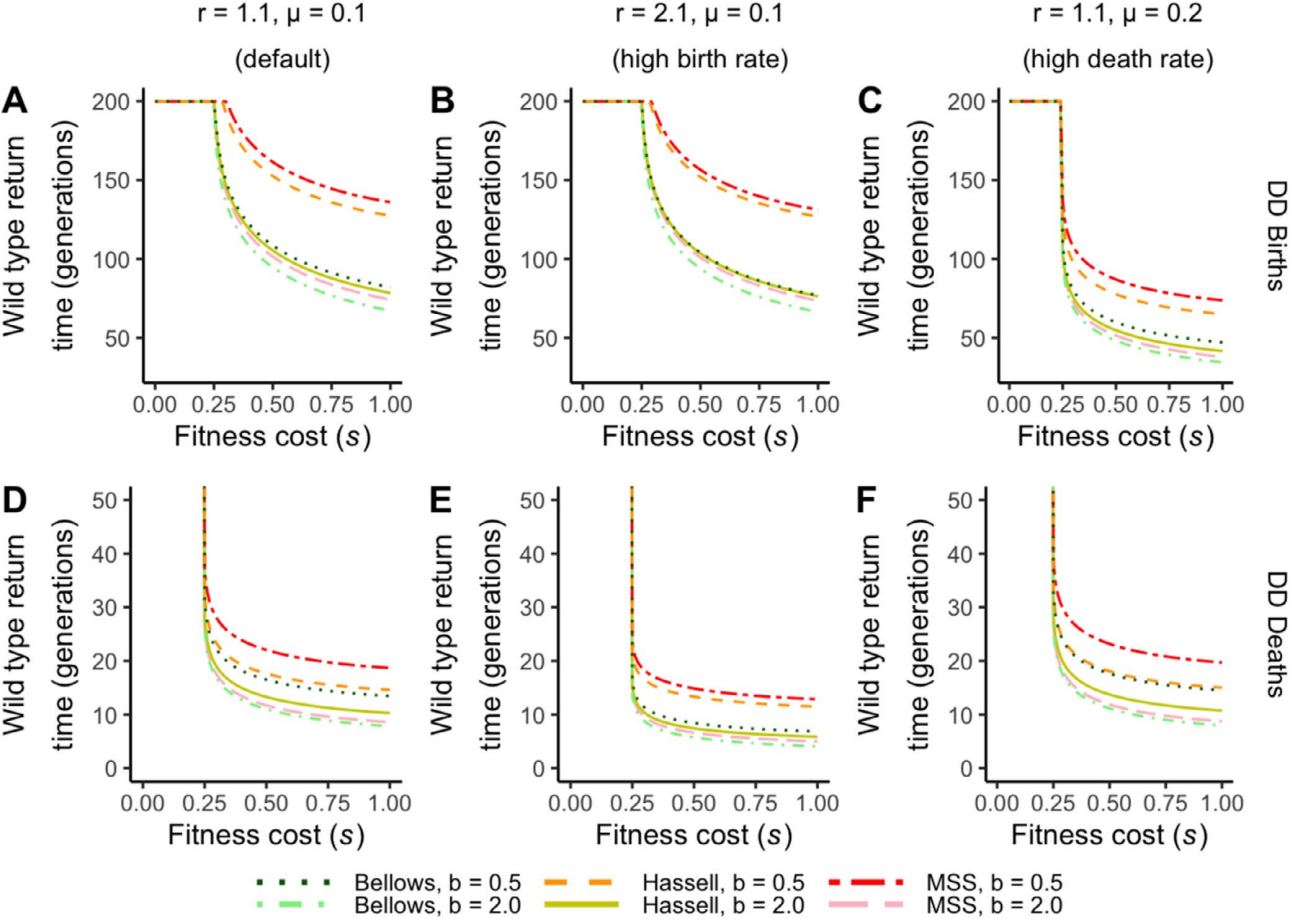
Analysis of drive efficacy in terms of wild‐type recovery time for density‐dependent models in births and deaths. The time for wild types to return above 99% of equilibrium, as shown in Figure 3C (density‐dependent births) and 3F (density‐dependent deaths) is analysed in three scenarios under (A, D) default birth and death rates, (B, E) high birth rate (*r* = 2.1) and default death rate, (C, F) default birth rate and high death rate (*μ* = 0.2). The scenarios are presented for both density‐dependent births (A–C) and density‐dependent deaths (D–F).

For density‐dependent births, changes in the birth rate had little effect on the time taken for wild types to recover compared to control (Figure 7A,B). However, increasing the mortality rates significantly reduced the time taken for the wild types to recover to the equilibrium by approximately 50% (Figure 7C). This observation makes ecological sense, as a higher death rate leads to reduced longevity across all genotypes, resulting in more rapid population dynamics during each generation and consequently bringing the wild‐type return time forward. The relative time taken for each density‐dependent model to achieve wild‐type recovery remains unchanged across all three scenarios. For example, the undercompensating MSS model is consistently observed to lead to the longest time for wild types to return to equilibrium, while the overcompensating Bellows model always requires the fewest generations. Importantly, there is also no change in the fitness cost threshold required for drive individuals to establish due to variation in birth or death rates.

#### 3.5.2 Density‐Dependent Deaths: The Sensitivity of Ecological Parameters on Drive Efficacy

The sensitivity of wild‐type recovery time to variations in core ecological parameters was also investigated under density‐dependent mortality. In contrast to the findings from density‐dependent birth models, where results from the high birth rate scenario are comparable to the default, the high birth rate scenario in density‐dependent death models actually leads to a more rapid re‐establishment of wild types (Figure 7D, E). This re‐establishment was observed within 10 generations for all overcompensating models of density dependence. Furthermore, undercompensating Hassell density dependence, which behaves similarly to the undercompensating Bellows model in the default control scenario, leads to wild‐type recovery time similar to the most rapid undercompensating MSS model under the high birth rate scenario. Likewise, under the high birth rate scenario, the time required for wild‐type recovery for the undercompensating Bellows model closely resembles that of the overcompensating Hassell and other models. However, as observed with density‐dependent birth models, in all scenarios, none of the models alter the specific sequence of recovery times as outlined in Figure 3. On the other hand, a larger death rate in density‐dependent death models does not produce more rapid dynamics; instead, it results in wild‐type recovery times similar to those under default settings (Figure 7F).

## 4 Discussion

In this study, we have adapted and formulated a generic yet flexible mathematical model for analysing the role of density‐dependent intraspecific competitions in determining the effectiveness of high‐threshold two‐locus underdominance gene drive systems.

In summary, we found that density dependence plays a significant role in regulating the degree and speed at which population dynamics occur. This difference is observed between employing different density‐dependent functions but more predominantly, between processes of density dependence on births and deaths. Such influence could potentially have an impact on the efficacy of drive releases, especially when the underdominance gene drive design carries a significant fitness penalty. On the other hand, neither the strength nor type of density‐dependent feedback mechanisms directly influence the fitness cost threshold for drive establishment. Our findings are a novel extension and in agreement with the previous work by Edgington & Alphey on two‐locus underdominance systems with a fixed fitness penalty of 0.05 per construct, where they highlighted that while density dependence significantly influences the population dynamics, it does not ultimately determine the outcome of a given control strategy (Edgington and Alphey 2018). Subsequent analyses further indicate that this fitness threshold remains independent of density‐dependent competitions when more complex ecological conditions, such as the dominance of fitness cost, *h* or asymmetric fitness costs for the drive construct, are considered. Given our limited knowledge of population regulation in vector species, our findings highlight that different density‐dependent feedbacks can lead to a range of possible outcomes for underdominance drive biocontrol methods. The details of these outcomes are discussed below.

First, the mathematical functions that give rise to different density‐dependent dynamics were assessed in terms of their effects on gene drive efficacy. Three independent functions of density dependence were considered: that of Bellows, Hassell and Maynard Smith‐Slatkin. Despite their similar forms and close mathematical relationships, subtle differences were observed in their implications for population dynamics and, subsequently, gene drive efficacy. This is especially true when we further compare their regulations in density‐dependent births and deaths. For example, the Hassell and MSS models had similar levels of population suppression upon increasing fitness costs when acting on births, but the two models are more significantly different when the population experiences density‐dependent deaths. Similarly, the Bellows model is generally more gradual, but under certain conditions can be comparable to the Hassell function (undercompensating density‐dependent deaths). These diverse outcomes suggest that the specific form of density dependence should be a consideration when developing drive release strategies, as the level of population suppression would have an effect on the introduction threshold frequency of gene drives. For instance, in a scenario when migration is considered between populations, the possibilities of wild‐type recolonisation would be determined by the extent of local population suppression as a result of density‐dependent regulations. Greater suppression of local individuals would make the recovery of wild types more likely, as it effectively lowers the frequency of drive individuals. This type of dynamics is similar to the ‘chasing’ effects characterised in previous modelling studies on suppression drives (Birand et al. 2022; Champer et al. 2021). Furthermore, if the drive frequency falls below the introduction threshold frequency after chasing, then it would not succeed in further establishment. This outcome is more likely under weaker density‐dependent processes (such as the undercompensating MSS function) as greater population suppression is found under this condition. However, for disease vectors or agricultural pests, difficulties lie in finding the best‐fit model describing density dependence, as we have little understanding of the relevant ecological feedback in these systems.

The strength of ecological intraspecific competition, portrayed by the parameter *b*, also plays an important role in relation to the density‐dependent functions. Our study considered two strengths of density dependence: contest‐like undercompensating/perfectly‐compensating competition and scramble‐like overcompensation where resources are equally distributed to accommodate a range of possible ecological scenarios for various target species for gene drive biocontrol. For instance, the larval stages of *Anopheles gambiae* are believed to experience contest competitions (Gimnig et al. 2002; Muriu et al. 2013; White et al. 2011), while the olive fruit fly, *Bactrocera oleae* , seems to engage in scramble competitions (Burrack et al. 2009). On the other hand, cohorts of *Aedes albopictus* showed contest competitions but females displayed scramble responses separately (Gavotte et al. 2009). Leaving aside the question of which density‐dependent function is most appropriate for each species, crucially, with undercompensating dynamics that most insects hence vector targets experience (Hassell, Lawton, and May 1976), the results indicate a greater effect of population suppression from drive genetics. This is because the population has reduced abilities to recover from a weaker form of density‐dependent feedback. As is the case for accounting different density‐dependent models, we argue that the strength of density dependence should be carefully considered in drive designs and studies. Since *b* is an exponent in density‐dependent models, its effects would be wide‐ranging. A closer estimate for under‐and‐overcompensating dynamics (beyond our simplified two‐value approach) would therefore be necessary to account more accurately for scenarios of species‐specific release.

Lastly, a sensitivity analysis was conducted to demonstrate the possible effects from variations in birth and death rates, since wide‐ranging values may be applied beyond this non‐specific model. This analysis is particularly crucial for our density‐dependent death model, as the effects from density‐dependent competition are incorporated as an addition (rather than multiplication, as in density‐dependent births) to the death rate to prevent an unrealistic zero initial death rate. However, this assumption comes with the limitation that the size of density‐dependent functions is independent of the death rate. The relative size of death rates in relation to density‐dependent functions thus matters, and its significance is explored here. Results demonstrate that for density‐dependent birth models, the population dynamics proceed faster when the base death rate is increased. Simultaneously, for density‐dependent death models, it is the increase in base birth rate that accelerates the speed of population dynamics. Our results corroborate the complexity in dealing with different types of density‐dependent feedback, as they are subject to change under different ecological scenarios and conditions (Hassell, Lawton, and May 1976; May 1975; Mueller and Joshi 2000). While future models may be extended to incorporate density‐dependent death functions both as an addition and multiplication on death to address potential drawbacks, our analysis suggests that no significant effect is observed from the aforementioned limitations.

Whilst we acknowledge the difficulties in obtaining empirical data on vector ecology (Dhole, Lloyd, and Gould 2020), gaining further insights, even from possibly small‐scale studies, would help to unravel the current limitations in our understanding of the density dependence of insect populations. For example, experiments could be conducted with varying densities to observe the effects of density changes on various demographic factors, such as population size, birth rate, death rate and development time (Murdoch 1970; Nicholson 1957). Here we strongly advocate such efforts to extend our understanding of intraspecific competitive effects as density dependence matters for gene drive efficacy.

Most models on gene drive studies have prioritised efficacy and are more focused as a proof of concept for gene drives rather than investigating their ecological implications (Frieß et al. 2023). Even for models that have investigated density dependence, the focus has been on whether, rather than how, density dependence affects gene drive efficacy. Here, we took an alternative approach for demonstrating the diverse effects of density dependence by comparing it in different mathematical forms. The genetic complexity arising from the two‐locus drive construct means that any analytical predictions may be challenging. Despite this, the wide‐ranging impacts observed from our simple model on an example threshold drive system can be a starting point for considering more complex scenarios.

As with any other mathematical model, this study is built with several underlying assumptions and simplifications. For instance, the value of the birth rate employed here only serves as an estimate and, in reality, may be subject to temporal and spatial variation (Godfray 2013). Moreover, the population dynamics of any target species will also have substantial age and stage structure (Backus and Delborne 2019).

Time‐lagged effects are not included in this study to achieve generality, meaning that no oscillation is observed in overcompensating dynamics. However, this assumption that the population growth rate would respond instantly to changes in density may not be entirely realistic. In fact, the relative position where density dependence takes place plays an important role in determining gene drive efficacy (Alphey and Bonsall 2014).

Our simple model framework can be readily extended to include these additional features: time lags, age/stage structure, migration or seasonality. It would also be interesting to look at other types of density‐dependent processes, such as Allee effects. For example, it is well established that there is positive density dependence in mosquito mating, especially in a small population, and this could be especially important in dry seasonal environments (Carballar‐Lejarazú 2023).

Furthermore, here we have only looked at density dependence as a result of intraspecific competition, with curves typically characterised by a bottom‐up (concave up) shape, at least in undercompensating dynamics. It would also be interesting to look at density‐dependent curves bearing a top‐down (concave down) shape as a result of predation, such as from the Janzen‐Connell hypothesis and its associated population dynamics (Mittelbach and McGill 2019).

In conclusion, this study has demonstrated that the population dynamics of two‐locus underdominance gene drives depend on the specific density‐dependent functions (and parameters chosen). Differences observed from various forms of density‐dependent regulation could help strengthen our understanding of the ecological compatibility and robustness of the two‐locus underdominance gene drive systems. However, given the ecological uncertainty demonstrated by density dependence and to inform on potentially safe and confined biological control strategies, future developments should carefully deliberate on the specific ecological backgrounds and consider spatial and stochastic effects for such high‐threshold gene drive releases.

## Conflicts of Interest

The authors declare no conflicts of interest.

## Supporting information


Appendix S1.

Figure S1.–S3.

## Data Availability

Data for this study are available at https://osf.io/strhu/.
