# Supplementary material for: Ecology of Gene Drives: The Role of Density‐Dependent Feedbacks on the Efficacy and Dynamics of Two‐Locus Underdominance Gene Drive Systems
Source: Evol Appl. 2025 Mar 6;18(3):e70079. doi: 10.1111/eva.70079 (PMC11885413; doi:10.1111/eva.70079)
Supplement: Supplementary file 1 — Appendix S1. [file EVA-18-e70079-s002.pdf]

Appendix

Ecology of Gene Drives:
the Role of Density-dependent Feedbacks on the Efficacy and Dynamics of Two-locus Underdominance Gene Drive Systems

Ziqian Xu and Michael B. Bonsall

Contents

I Dihybrid Crosses **2**

II Progeny equations **4**

III Mathematical Models of Density Dependence **5**

IV Density parameter a **6**

I Dihybrid Crosses

In a 2-locus, 2-allele diploid system involving alleles A and a at one locus, and B and b at another, there are 81 possible genotype combinations among the progeny under Mendelian segregation. These dihybrid crosses, determined using Punnett Squares, are visualised in the following table. Here, N_i denotes the nine distinct genotypes, ranging from N_1 (AABB) to N_9 (aabb).

	$AABB$	$AABb$	$AAbb$	$AaBB$	$AaBb$	$Aabb$	$aaBB$	$aaBb$	$aabb$
$AABB \times AABB$	N_1^2	0	0	0	0	0	0	0	0
$AABB \times AABb$	$\frac{2N_1N2}{2}$	$\frac{2N_1N_2}{2}$	0	0	0	0	0	0	0
$AABB \times AAbb$	0	$2N_1N_3$	0	0	0	0	0	0	0
$AABB \times AaBB$	$\frac{2N_1N4}{2}$	0	0	$\frac{2N_1N4}{2}$	0	0	0	0	0
$AABB \times AaBb$	$\frac{2N_1N5}{4}$	$\frac{2N_1N5}{4}$	0	$\frac{2N_1N5}{4}$	$\frac{2N_1N5}{4}$	0	0	0	0
$AABB \times Aabb$	0	$\frac{2N_1N6}{2}$	0	0	$\frac{2N_1N6}{2}$	0	0	0	0
$AABB \times aaBB$	0	0	0	$2N_1N_7$	0	0	0	0	0
$AABB \times aaBb$	0	0	0	$\frac{2N_1N8}{2}$	$\frac{2N_1N8}{2}$	0	0	0	0
$AABB \times aabb$	0	0	0	0	$2N_1N_9$	0	0	0	0
$AABb \times AABb$	$\frac{N_2^2}{4}$	$\frac{N_2^2}{2}$	$\frac{N_2^2}{4}$	0	0	0	0	0	0
$AABb \times AAbb$	0	$\frac{2N_2N_3}{2}$	$\frac{2N_2N_3}{2}$	0	0	0	0	0	0
$AABb \times AaBB$	$\frac{2N_2N_4}{4}$	$\frac{2N_2N_4}{4}$	0	$\frac{2N_2N_4}{4}$	$\frac{2N_2N_4}{4}$	0	0	0	0
$AABb \times AaBb$	$\frac{2N_2N_5}{8}$	$\frac{2N_2N_5}{4}$	$\frac{2N_2N_5}{8}$	$\frac{2N_2N_5}{8}$	$\frac{2N_2N_5}{4}$	$\frac{2N_2N_5}{8}$	0	0	0
$AABb \times Aabb$	0	$\frac{2N_2N_6}{4}$	$\frac{2N_2N_6}{4}$	0	$\frac{2N_2N_6}{4}$	$\frac{2N_2N_6}{4}$	0	0	0
$AABb \times aaBB$	0	0	0	$\frac{2N_2N_7}{2}$	$\frac{2N_2N_7}{2}$	0	0	0	0
$AABb \times aaBb$	0	0	0	$\frac{2N_2N_8}{4}$	$\frac{2N_2N_8}{2}$	$\frac{2N_2N_8}{4}$	0	0	0
$AABb \times aabb$	0	0	0	0	$\frac{2N_2N_9}{2}$	$\frac{2N_2N_9}{2}$	0	0	0

	$AABB$	$AABb$	$AAbb$	$AaBB$	$AaBb$	$Aabb$	$aaBB$	$aaBb$	$aabb$
$AAbb \times AAbb$	0	0	N_3^2	0	0	0	0	0	0
$AAbb \times AaBB$	0	$\frac{2N_3N_4}{2}$	0	0	$\frac{2N_3N_4}{2}$	0	0	0	0
$AAbb \times AaBb$	0	$\frac{2N_3N_5}{4}$	$\frac{2N_3N_5}{4}$	0	$\frac{2N_3N_5}{4}$	$\frac{2N_3N_5}{4}$	0	0	0
$AAbb \times Aabb$	0	0	$\frac{2N_3N_6}{2}$	0	0	$\frac{2N_3N_6}{2}$	0	0	0
$AAbb \times aaBB$	0	0	0	0	$2N_3N_7$	0	0	0	0
$AAbb \times aaBb$	0	0	0	0	$\frac{2N_3N_8}{2}$	$\frac{2N_3N_8}{2}$	0	0	0
$AAbb \times aabb$	0	0	0	0	0	$2N_3N_9$	0	0	0
$AaBB \times AaBB$	$\frac{N_4^2}{4}$	0	0	$\frac{N_4^2}{2}$	0	0	$\frac{N_4^2}{4}$	0	0
$AaBB \times AaBb$	$\frac{2N_4N_5}{8}$	$\frac{2N_4N_5}{8}$	0	$\frac{2N_4N_5}{4}$	$\frac{2N_4N_5}{4}$	0	$\frac{2N_4N_5}{8}$	$\frac{2N_4N_5}{8}$	0
$AaBB \times Aabb$	0	$\frac{2N_4N_6}{4}$	0	0	$\frac{2N_4N_6}{2}$	0	0	$\frac{2N_4N_6}{4}$	0
$AaBB \times aaBB$	0	0	0	$\frac{2N_4N_7}{2}$	0	0	$\frac{2N_4N_7}{2}$	0	0
$AaBB \times aaBb$	0	0	0	$\frac{2N_4N_8}{4}$	$\frac{2N_4N_8}{4}$	0	$\frac{2N_4N_8}{4}$	$\frac{2N_4N_8}{4}$	0
$AaBB \times aabb$	0	0	0	0	$\frac{2N_4N_9}{2}$	0	0	$\frac{2N_4N_9}{2}$	0
$AaBb \times AaBb$	$\frac{N_5^2}{16}$	$\frac{N_5^2}{8}$	$\frac{N_5^2}{16}$	$\frac{N_5^2}{8}$	$\frac{N_5^2}{4}$	$\frac{N_5^2}{8}$	$\frac{N_5^2}{16}$	$\frac{N_5^2}{8}$	$\frac{N_5^2}{16}$
$AaBb \times Aabb$	0	$\frac{2N_5N_6}{8}$	$\frac{2N_5N_6}{8}$	0	$\frac{2N_5N_6}{4}$	$\frac{2N_5N_6}{4}$	0	$\frac{2N_5N_6}{8}$	$\frac{2N_5N_6}{8}$
$AaBb \times aaBB$	0	0	0	$\frac{2N_5N_7}{4}$	$\frac{2N_5N_7}{4}$	0	$\frac{2N_5N_7}{4}$	$\frac{2N_5N_7}{4}$	0
$AaBb \times aaBb$	0	0	0	$\frac{2N_5N_8}{8}$	$\frac{2N_5N_8}{4}$	$\frac{2N_5N_8}{8}$	$\frac{2N_5N_8}{8}$	$\frac{2N_5N_8}{4}$	$\frac{2N_5N_8}{8}$
$AaBb \times aabb$	0	0	0	0	$\frac{2N_5N_9}{4}$	$\frac{2N_5N_9}{4}$	0	$\frac{2N_5N_9}{4}$	$\frac{2N_5N_9}{4}$
$Aabb \times Aabb$	0	0	$\frac{N_6^2}{4}$	0	0	$\frac{N_6^2}{2}$	0	0	$\frac{N_6^2}{4}$
$Aabb \times aaBB$	0	0	0	0	$\frac{2N_6N_7}{2}$	0	0	$\frac{2N_6N_7}{2}$	0
$Aabb \times aaBb$	0	0	0	0	$\frac{2N_6N_8}{4}$	$\frac{2N_6N_8}{4}$	0	$\frac{2N_6N_8}{4}$	$\frac{2N_6N_8}{4}$
$Aabb \times aabb$	0	0	0	0	0	$\frac{2N_6N_9}{2}$	0	0	$\frac{2N_6N_9}{2}$
$aaBB \times aaBB$	0	0	0	0	0	0	N_7^2	0	0
$aaBB \times aaBb$	0	0	0	0	0	0	$\frac{2N_7N_8}{2}$	$\frac{2N_7N_8}{2}$	0
$aaBB \times aabb$	0	0	0	0	0	0	0	$2N_7N_9$	0
$aaBb \times aaBb$	0	0	0	0	0	0	$\frac{N_8^2}{4}$	$\frac{N_8^2}{2}$	$\frac{N_8^2}{4}$
$aaBb \times aabb$	0	0	0	0	0	0	0	$\frac{2N_8N_9}{2}$	$\frac{2N_8N_9}{2}$
$aabb \times aabb$	0	0	0	0	0	0	0	0	N_9^2

II Progeny equations

Progeny equations may be formulated using results from the previous section, which determines the population genetics in this model:

$$\nu_1(t) = \frac{1}{N}\left(N_1^2 + N_1N_2 + N_1N_4 + \frac{N_1N_5}{2} + \frac{N_2^2}{4} + \frac{N_2N_4}{2} + \frac{N_2N_5}{4} + \frac{N_4^2}{4} + \frac{N_4N_5}{4} + \frac{N_5^2}{16}\right) \tag{1}$$

$$\begin{aligned}\nu_2(t) = \frac{1}{N}\Bigg(&N_1N_2 + 2N_1N_3 + \frac{N_1N_5}{2} + N_1N_6 + \frac{N_2^2}{2} + N_2N_3 + \frac{N_2N_4}{2} + \frac{N_2N_5}{2} + \frac{N_2N_6}{2} + N_3N_4 \\ &+ \frac{N_3N_5}{2} + \frac{N_4N_5}{4} + \frac{N_4N_6}{2} + \frac{N_5^2}{8} + \frac{N_5N_6}{4}\Bigg)\end{aligned} \tag{2}$$

$$\nu_3(t) = \frac{1}{N}\left(\frac{N_2^2}{4} + N_2N_3 + \frac{N_2N_5}{4} + \frac{N_2N_6}{2} + N_3^2 + \frac{N_3N_5}{2} + N_3N_6 + \frac{N_5^2}{16} + \frac{N_5N_6}{4} + \frac{N_6^2}{4}\right) \tag{3}$$

$$\begin{aligned}\nu_4(t) = \frac{1}{N}\Bigg(&N_1N_4 + \frac{N_1N_5}{2} + 2N_1N_7 + N_1N_8 + \frac{N_2N_4}{2} + \frac{N_2N_5}{4} + N_2N_7 + \frac{N_2N_8}{2} + \frac{N_4^2}{2} + \frac{N_4N_5}{2} \\ &+ N_4N_7 + \frac{N_4N_8}{2} + \frac{N_5^2}{8} + \frac{N_5N_7}{2} + \frac{N_5N_8}{4}\Bigg)\end{aligned} \tag{4}$$

$$\begin{aligned}\nu_5(t) = \frac{1}{N}\Bigg(&\frac{N_1N_5}{2} + N_1N_6 + N_1N_8 + 2N_1N_9 + \frac{N_2N_4}{2} + \frac{N_2N_5}{2} + \frac{N_2N_6}{2} + N_2N_7 + N_2N_8 + N_2N_9 + N_3N_4 \\ &+ \frac{N_3N_5}{2} + 2N_3N_7 + N_3N_8 + \frac{N_4N_5}{2} + N_4N_6 + \frac{N_4N_8}{2} + N_4N_9 + \frac{N_5^2}{4} + \frac{N_5N_6}{2} + \frac{N_5N_7}{2} + \frac{N_5N_8}{2} \\ &+ \frac{N_5N_9}{2} + N_6N_7 + \frac{N_6N_8}{2}\Bigg)\end{aligned} \tag{5}$$

$$\begin{aligned}\nu_6(t) = \frac{1}{N}\Bigg(&\frac{N_2N_5}{4} + \frac{N_2N_6}{2} + \frac{N_2N_8}{2} + N_2N_9 + \frac{N_3N_5}{2} + N_3N_6 + N_3N_8 + 2N_3N_9 + \frac{N_5^2}{8} + \frac{N_5N_6}{2} \\ &+ \frac{N_5N_8}{4} + \frac{N_5N_9}{2} + \frac{N_6^2}{2} + \frac{N_6N_8}{2} + N_6N_9\Bigg)\end{aligned} \tag{6}$$

$$\nu_7(t) = \frac{1}{N}\left(\frac{N_4^2}{4} + \frac{N_4N_5}{4} + N_4N_7 + \frac{N_4N_8}{2} + \frac{N_5^2}{16} + \frac{N_5N_7}{2} + \frac{N_5N_8}{4} + N_7^2 + N_7N_8 + \frac{N_8^2}{4}\right) \tag{7}$$

$$\begin{aligned}\nu_8(t) = \frac{1}{N}\Bigg(&\frac{N_4N_5}{4} + \frac{N_4N_6}{2} + \frac{N_4N_8}{2} + N_4N_9 + \frac{N_5^2}{8} + \frac{N_5N_6}{4} + \frac{N_5N_7}{2} + \frac{N_5N_8}{2} + \frac{N_5N_9}{2} + N_6N_7 \\ &+ \frac{N_6N_8}{2} + N_7N_8 + 2N_7N_9 + \frac{N_8^2}{2} + N_8N_9\Bigg)\end{aligned} \tag{8}$$

$$\nu_9(t) = \frac{1}{N}\left(\frac{N_5^2}{16} + \frac{N_5N_6}{4} + \frac{N_5N_8}{4} + \frac{N_5N_9}{2} + \frac{N_6^2}{4} + \frac{N_6N_8}{2} + N_6N_9 + \frac{N_8^2}{4} + N_8N_9 + N_9^2\right) \tag{9}$$

where

$$N = \sum_{n=1}^{9} N_i$$

III Mathematical Models of Density Dependence

General form of model for density-dependent birth regulations:

$$\frac{dN_i}{dt} = r\nu_i(t)\psi_i f(N) - \mu N_i(t)$$

General form of model for density-dependent death regulations:

$$\frac{dN_i}{dt} = r\nu_i(t)\psi_i - (\mu + f(N))N_i(t)$$

where r is the birth rate, μ is the death rate, ν_i represents progeny, ψ_i represents the relative fitness of each genotype, and $f(N)$ represents the density-dependent functions as outlined by Bellows (1981):

Entry	Authors	Density-dependent Birth	Density-dependent Death
1	Skellam (1951)	$(1 + aN)^{-1}$	$\ln(1 + aN)$
2	Maynard Smith & Slatkin (1973)	$(1 + (aN)^b)^{-1}$	$\ln(1 + (aN)^b)$
3	Hassell (1975)	$(1 + aN)^{-b}$	$b\ln(1 + aN)$
4	Bellows (1981)	$\exp(-aN^b)$	aN^b

aN in this table denotes the sum population of all genotypes, i.e. $\sum_{j=1}^{9} a\nu_j(t)$ in the models.

III.1 Density-dependent births

III.1.1 Skellam (1951)

$$\frac{dN_i}{dt} = \frac{r\nu_i(t)\psi_i}{1 + \left(\sum_{j=1}^{9} a\nu_j(t)\right)} - \mu N_i(t)$$

III.1.2 Maynard Smith & Slatkin (1973)

$$\frac{dN_i}{dt} = \frac{r\nu_i(t)\psi_i}{1 + \left(\sum_{j=1}^{9} a\nu_j(t)\right)^b} - \mu N_i(t)$$

III.1.3 Hassell (1975)

$$\frac{dN_i}{dt} = \frac{r\nu_i(t)\psi_i}{\left(1 + \sum_{j=1}^{9} a\nu_j(t)\right)^b} - \mu N_i(t)$$

III.1.4 Bellows (1981)

$$\frac{dN_i}{dt} = \frac{r\nu_i(t)\psi_i}{e^{\left(\sum_{j=1}^{9} a\nu_j(t)\right)^b}} - \mu N_i(t)$$

III.2 Density-dependent deaths

III.2.1 Skellam (1951)

$$\frac{dN_i}{dt} = r\nu_i(t)\psi_i - (\mu + \ln(1 + \left(\sum_{j=1}^{9} a\nu_j(t)\right)))N_i(t)$$

III.2.2 Maynard Smith & Slatkin (1973)

$$\frac{dN_i}{dt} = r\nu_i(t)\psi_i - (\mu + \ln(1 + \left(\sum_{j=1}^{9} a\nu_j(t)\right)^b))N_i(t)$$

III.2.3 Hassell (1975)

$$\frac{dN_i}{dt} = r\nu_i(t)\psi_i - (\mu + b\ln(1 + \left(\sum_{j=1}^{9} a\nu_j(t)\right)))N_i(t)$$

III.2.4 Bellows (1981)

$$\frac{dN_i}{dt} = r\nu_i(t)\psi_i - (\mu + \left(\sum_{j=1}^{9} a\nu_j(t)\right)^b)N_i(t)$$

IV Density parameter a

The density parameter a is adjusted in density-dependent functions to ensure that the steady-state population equilibrium, denoted N^*, is fixed at 10,000. The specific value of a for each function is determined as follows:

IV.1 Density-dependent births

IV.1.1 Skellam (1951)

$$a = \frac{1}{N^*}(\frac{r}{\mu} - 1)$$

IV.1.2 Maynard Smith & Slatkin (1973)

$$a = \frac{1}{N^*}\sqrt[b]{\frac{r}{\mu} - 1}$$

IV.1.3 Hassell (1975)

$$a = \frac{1}{N^*}(\sqrt[b]{\frac{r}{\mu}} - 1)$$

IV.1.4 Bellows (1981)

$$a = \frac{1}{{N^*}^b}\ln(\frac{r}{\mu})$$

IV.2 Density-dependent deaths

IV.2.1 Skellam (1951)

$$a = \frac{1}{N^*}(e^{r-\mu} - 1)$$

IV.2.2 Maynard Smith & Slatkin (1973)

$$a = \frac{1}{N^*}\sqrt[b]{e^{r-\mu} - 1}$$

IV.2.3 Hassell (1975)

$$a = \frac{1}{N^*}(e^{\frac{r-\mu}{b}} - 1)$$

IV.2.4 Bellows (1981)

$$a = \frac{1}{{N^*}^b}(r - \mu)$$
