# Supplementary material for: Ecology of Gene Drives: The Role of Density‐Dependent Feedbacks on the Efficacy and Dynamics of Two‐Locus Underdominance Gene Drive Systems
Source: Evol Appl. 2025 Mar 6;18(3):e70079. doi: 10.1111/eva.70079 (PMC11885413; doi:10.1111/eva.70079)
Supplement: Supplementary file 2 — Figure S1.–S3. [file EVA-18-e70079-s001.pdf]

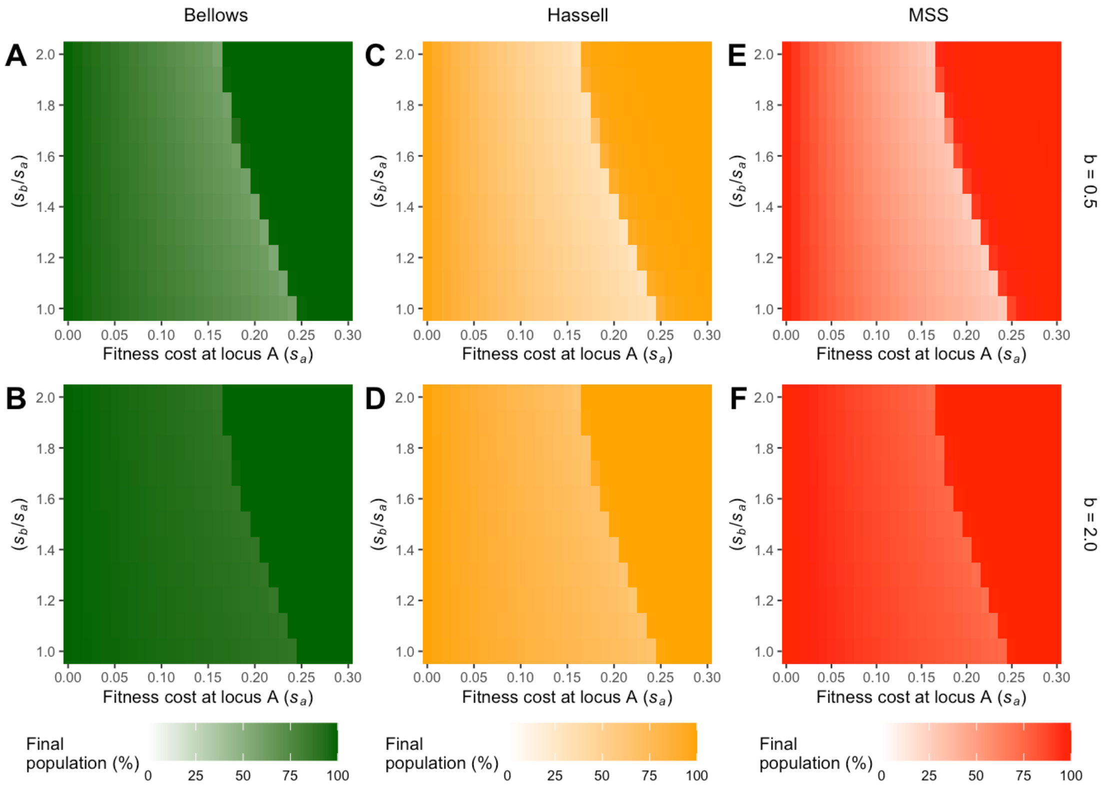


**Supplementary Figure 1. Heatmaps for drive efficacy analysis of varying fitness costs (*s*) of density-dependent birth models without assuming equal fitness costs at the two loci**. The fitness cost ratio between the two loci ($s_b/s_a$) is plotted against the fitness cost at locus A ($s_a$), while the final total population percentage is indicated by the colour gradient: minima are coloured in white, while maxima are coloured in green, orange and red for Bellows (A, B), Hassell (C, D) and MSS (E, F) models, respectively. Figures in the top panel (A, C, E) are for undercompensating dynamics at $b$ = 0.5 and figures in the bottom panel (B, D, F) are for overcompensating dynamics at $b$ = 2.0. Simulations were run across the full range of $s_a$ from 0 to 0.5, with an increment of 0.01, but results are displayed only up to 0.30 in this figure.

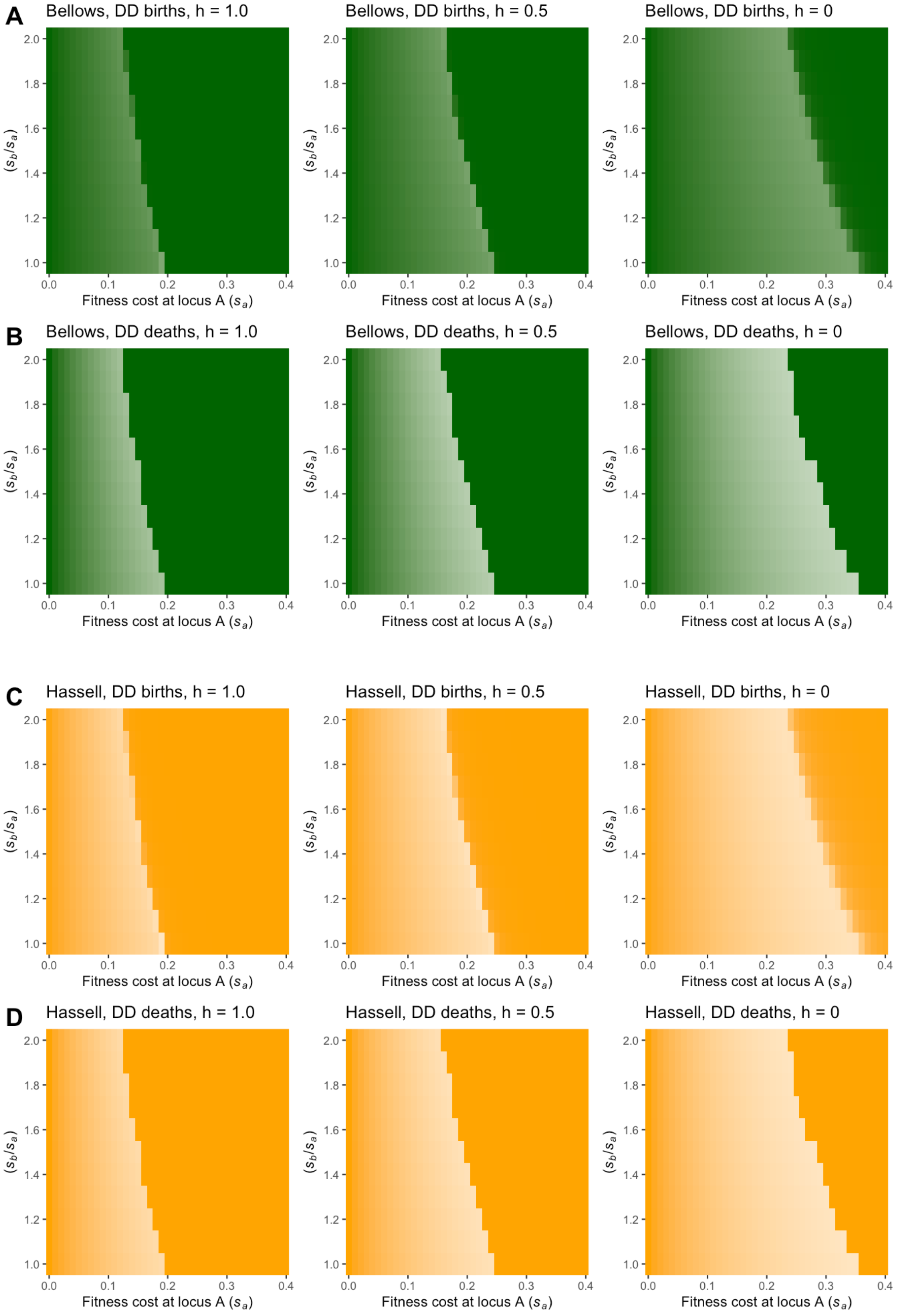


**Supplementary Figure 2. Heatmaps for drive efficacy analysis of varying fitness costs (*s*) of undercompensating Bellows and Hassell density-dependent models in three conditions of dominance of fitness cost (*h*) without assuming equal**

**fitness costs at the two loci**. Here, heatmaps in Figure 6 are performed under three scenarios of dominance of fitness costs in heterozygotes (*h*): (i) when *h* is fully dominant at 1.0, (ii) when *h* is default at 0.5, (iii) when *h* is fully recessive at 0, and when the density dependence acts on (A, C) births, and (B, D) deaths. The percentage of the final total population is indicated by the colour gradient, where minima are coloured in white, and maxima are coloured in green and orange for Bellows (A, B) and Hassell (C, D) models, respectively. Simulations were run across the full range of $s_a$ from 0 to 0.5, with an increment of 0.01, but results are displayed only up to 0.40 in the figures.

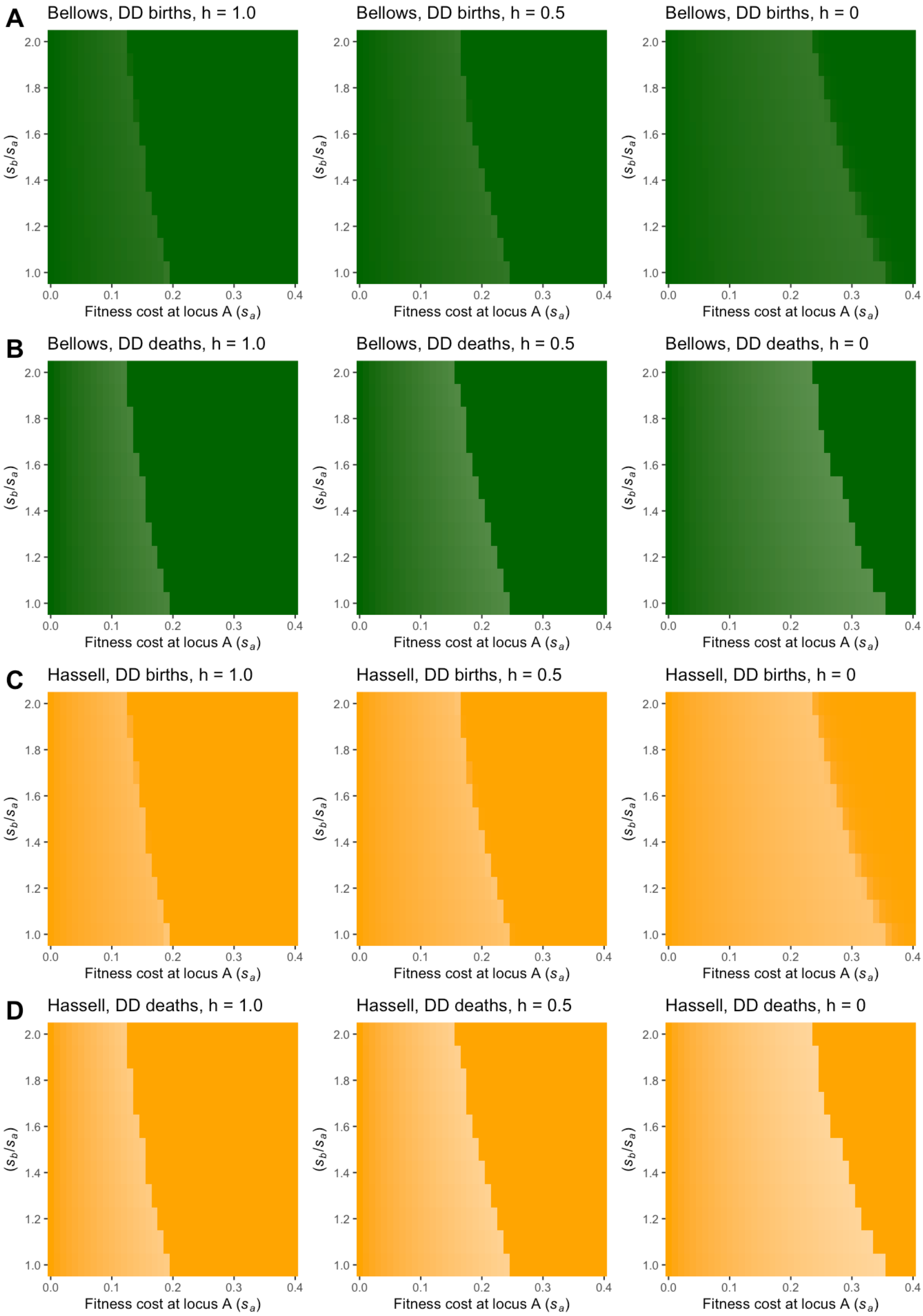
A
Bellows, DD births, h = 1.0
Bellows, DD births, h = 0.5
Bellows, DD births, h = 0
B
Bellows, DD deaths, h = 1.0
Bellows, DD deaths, h = 0.5
Bellows, DD deaths, h = 0
C
Hassell, DD births, h = 1.0
Hassell, DD births, h = 0.5
Hassell, DD births, h = 0
D
Hassell, DD deaths, h = 1.0
Hassell, DD deaths, h = 0.5
Hassell, DD deaths, h = 0
$(s_b/s_a)$
1.0 1.2 1.4 1.6 1.8 2.0
0.0 0.1 0.2 0.3 0.4
Fitness cost at locus A ($s_a$)

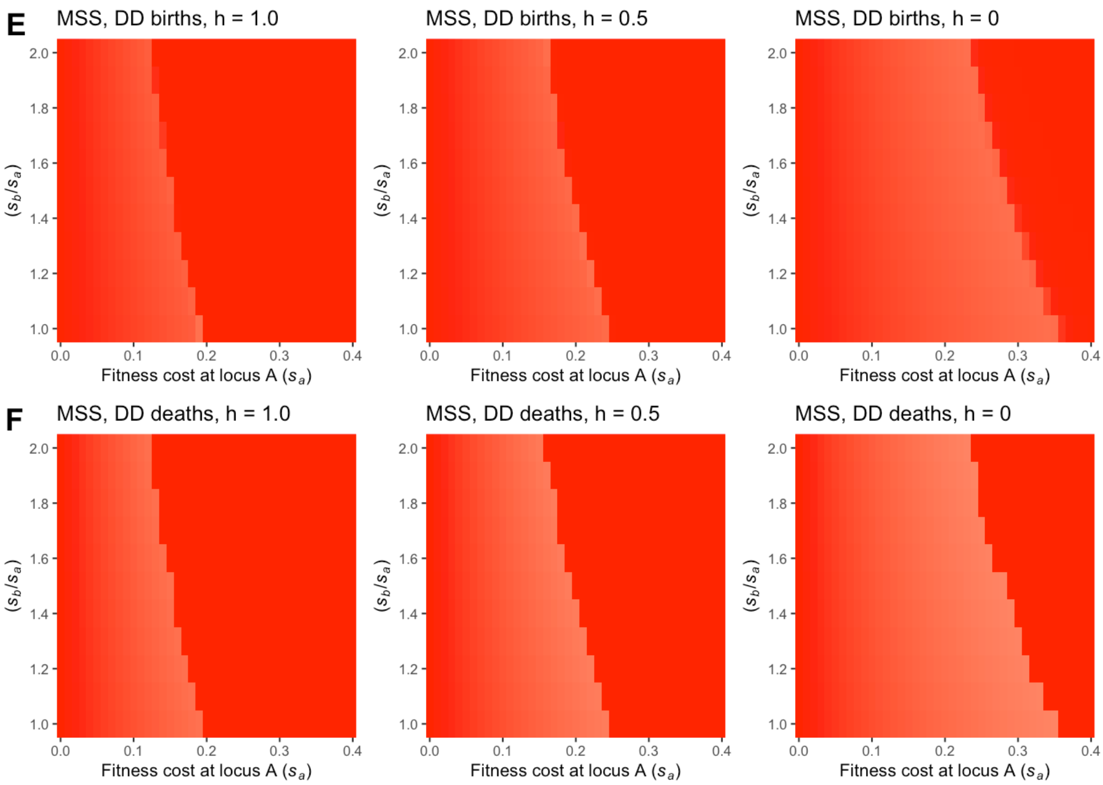


**Supplementary Figure 3. Heatmaps for drive efficacy analysis of varying fitness costs (*s*) of overcompensating density-dependent models in three conditions of dominance of fitness cost (*h*) without assuming equal fitness costs at the two loci**. Here, heatmaps in Figure 6 are performed under three scenarios of dominance of fitness costs in heterozygotes (*h*): (i) when *h* is fully dominant at 1.0, (ii) when *h* is default at 0.5, (iii) when *h* is fully recessive at 0, and when the density dependence acts on (A, C, E) births, and (B, D, F) deaths. The percentage of the final total population is indicated by the colour gradient where minima are coloured in white, and maxima are coloured in green, orange and red for Bellows (A, B), Hassell (C, D) and MSS (E, F) models, respectively. Simulations were run across the full of $s_a$ from 0 to 0.5, with an increment of 0.01, but results are displayed only up to 0.40 in the figures.
